# Spatial heterogeneity of peri-tumoural lipid composition in postmenopausal patients with oestrogen receptor positive breast cancer

**DOI:** 10.1038/s41598-024-55458-y

**Published:** 2024-02-26

**Authors:** Sai Man Cheung, Kwok-Shing Chan, Wenshu Zhou, Ehab Husain, Tanja Gagliardi, Yazan Masannat, Jiabao He

**Affiliations:** 1 School of Medicine, Medical Sciences and Nutrition, University of Aberdeen, Aberdeen, UK; 2 Athinoula A. Martinos Center for Biomedical Imaging, Charlestown, MA USA; 3 Department of Radiology, Harvard Medical School, Boston, MA USA; 4 Department of Pathology, Aberdeen Royal Infirmary, Aberdeen, UK; 5 Department of Radiology, Royal Marsden Hospital, London, UK; 6 Broomfield Breast Unit, Broomfield Hospital, Mid and South Essex NHS Trust, Chelmsford, UK; 7 London Breast Institute, Princess Grace Hospital, London, UK; 8 Faculty of Medical Sciences, Newcastle Magnetic Resonance Centre, Translational and Clinical Research Institute, Newcastle University, Newcastle upon Tyne, UK

**Keywords:** Lipid composition, Heterogeneity, Oestrogen receptor, Inflammation, Breast cancer, Medical research, Oncology

## Abstract

Deregulation of lipid composition in adipose tissue adjacent to breast tumour is observed in ex vivo and animal models. Novel non-invasive magnetic resonance imaging (MRI) allows rapid lipid mapping of the human whole breast. We set out to elucidate the spatial heterogeneity of peri-tumoural lipid composition in postmenopausal patients with oestrogen receptor positive (ER +) breast cancer. Thirteen participants (mean age, 62 ± [SD] 6 years) with ER + breast cancer and 13 age-matched postmenopausal healthy controls were scanned on MRI. The number of double bonds in triglycerides was computed from MRI images to derive lipid composition maps of monounsaturated, polyunsaturated, and saturated fatty acids (MUFA, PUFA, SFA). The spatial heterogeneity measures (mean, median, skewness, entropy and kurtosis) of lipid composition in the peri-tumoural region and the whole breast of participants and in the whole breast of controls were computed. The Ki-67 proliferative activity marker and CD163 antibody on tumour-associated macrophages were assessed histologically. Mann Whitney *U* or Wilcoxon tests and Spearman’s coefficients were used to assess group differences and correlations, respectively. For comparison against the whole breast in participants, peri-tumoural MUFA had a lower mean (median (IQR), 0.40 (0.02), *p* < .001), lower median (0.42 (0.02), *p* < .001), a negative skewness with lower magnitude (− 1.65 (0.77), *p* = .001), higher entropy (4.35 (0.64), *p* = .007) and lower kurtosis (5.13 (3.99), *p* = .001). Peri-tumoural PUFA had a lower mean (*p* < .001), lower median (*p* < .001), a positive skewness with higher magnitude (*p* = .005) and lower entropy (*p* = .002). Peri-tumoural SFA had a higher mean (*p* < .001), higher median (*p* < .001), a positive skewness with lower magnitude (*p* < .001) and lower entropy (*p* = .012). For comparison against the whole breast in controls, peri-tumoural MUFA had a negative skewness with lower magnitude (*p* = .01) and lower kurtosis (*p* = .009), however there was no difference in PUFA or SFA. CD163 moderately correlated with peri-tumoural MUFA skewness (*r*_*s*_ = − .64), PUFA entropy (*r*_*s*_ = .63) and SFA skewness (*r*_*s*_ = .59). There was a lower MUFA and PUFA while a higher SFA, and a higher heterogeneity of MUFA while a lower heterogeneity of PUFA and SFA, in the peri-tumoural region in comparison with the whole breast tissue. The degree of lipid deregulation was associated with inflammation as indicated by CD163 antibody on macrophages, serving as potential marker for early diagnosis and response to therapy.

## Introduction

Oestrogen receptor-positive (ER +) breast cancer constitutes more than two-thirds of all postmenopausal cases^1^, which account for 75% of all new diagnoses. While hormonal treatment has had high efficacy in women with postmenopausal ER + breast cancer^2^, the typical hormonal regime requires maintenance therapy for 5 years, with adverse effects including blood clots, stroke and disruption to sexual and gynecologic quality of life^3^. Hence, an approach to determine the efficacy of hormonal therapy may allow for more precise and targeted treatment.

Oestrogen is predominantly modulated by mammary adipocytes in postmenopausal women^4^, and it induces the production of stearoyl-coenzyme-A desaturase-1 in ER + breast carcinoma cells^5^. An imbalance in overall lipid composition within the breast has been observed in postmenopausal women with ER + breast cancer based on loosely defined regions of interest^6,7^. Intratumoural spatial heterogeneity from *q*-space imaging reflects the spatial distribution of histological cellularity^8^, while higher entropy (increased heterogeneity in spatial distribution)^9^ from dynamic contrast-enhanced (DCE) MRI is associated with worse survival outcomes in triple negative compared to ER + ^10^ or Luminal A^11^ breast cancer. Adipocyte-derived free fatty acids support cancer cell development through activation of fatty acid oxidation to meet energy demand under glucose-starved condition^12^. Subsequently, breast cancer cells cultivated with adipocyte-derived free fatty acids undergo a metabolic switch towards anaerobic glycolysis for adenosine triphosphate production, and the metabolic shift is associated with increased epithelial to mesenchymal transition for improved colonisation of distant sites^13^. Pro-inflammatory polyunsaturated fatty acids (PUFA) stimulate inflammation of the tumour microenvironment^14^, and the recruitment of aromatase-enriched tumour-associated macrophages enable oestrogen synthesis and enhance ER + breast cancer proliferation^15^. Further, accelerated biosynthesis of monounsaturated fatty acids (MUFA) substantially increases storage of triglycerides as lipid droplets, an event linked to tamoxifen resistance^16^. Hence, peri-tumoural spatial heterogeneity of lipid composition may demonstrate sensitivity in determining response to hormonal therapy in postmenopausal ER + breast cancer.

The quantitative mapping of lipid composition in breast adipose and fibroglandular tissue is thus highly desirable. However, conventional method of single-voxel spectroscopy is limited to a single spatial location^17^, and the spatially resolved method of chemical shift imaging demands substantial scan time with susceptibility to the inhomogeneity in the scanner magnetic field^18,19^. Chemical shift-encoded imaging (CSEI) is a recently proposed method to allow lipid composition mapping in the thigh^19^, abdomen^20^ and breast^21^ within a clinically acceptable time frame and quantification of MUFA, PUFA and saturated fatty acids (SFA) in adipose tissues. A recent ex vivo study using CSEI showed deregulation of lipid composition in peri-tumoural adipose tissues in breast cancer^21^.

Tumours with pro-angiogenic and pro-metastatic profile show high infiltration density of CD163 antibody on tumour-associated macrophages to produce significantly higher levels of anti-inflammatory cytokine interleukin-10^22^. We therefore hypothesised that the spatial heterogeneity of peri-tumoural lipid composition in postmenopausal patients with ER + breast cancer deviates from the whole breast lipid composition in healthy controls and is associated with inflammatory activities from histopathological analysis. To probe this hypothesis, we conducted a two-group cross-sectional prospective study examining the peri-tumoural spatial heterogeneity of lipid composition on maps acquired from MRI scans in postmenopausal patients with breast cancer in comparison with age-matched controls.

## Methods

This Health Insurance Portability and Accountability Act-compliant study, performed between September 2017 and June 2019, was approved by the North of Scotland Research Ethics Service (REC Reference: 16/NS/0077), and written informed consent was obtained from all participants.

### Participants

**Figure 1:**
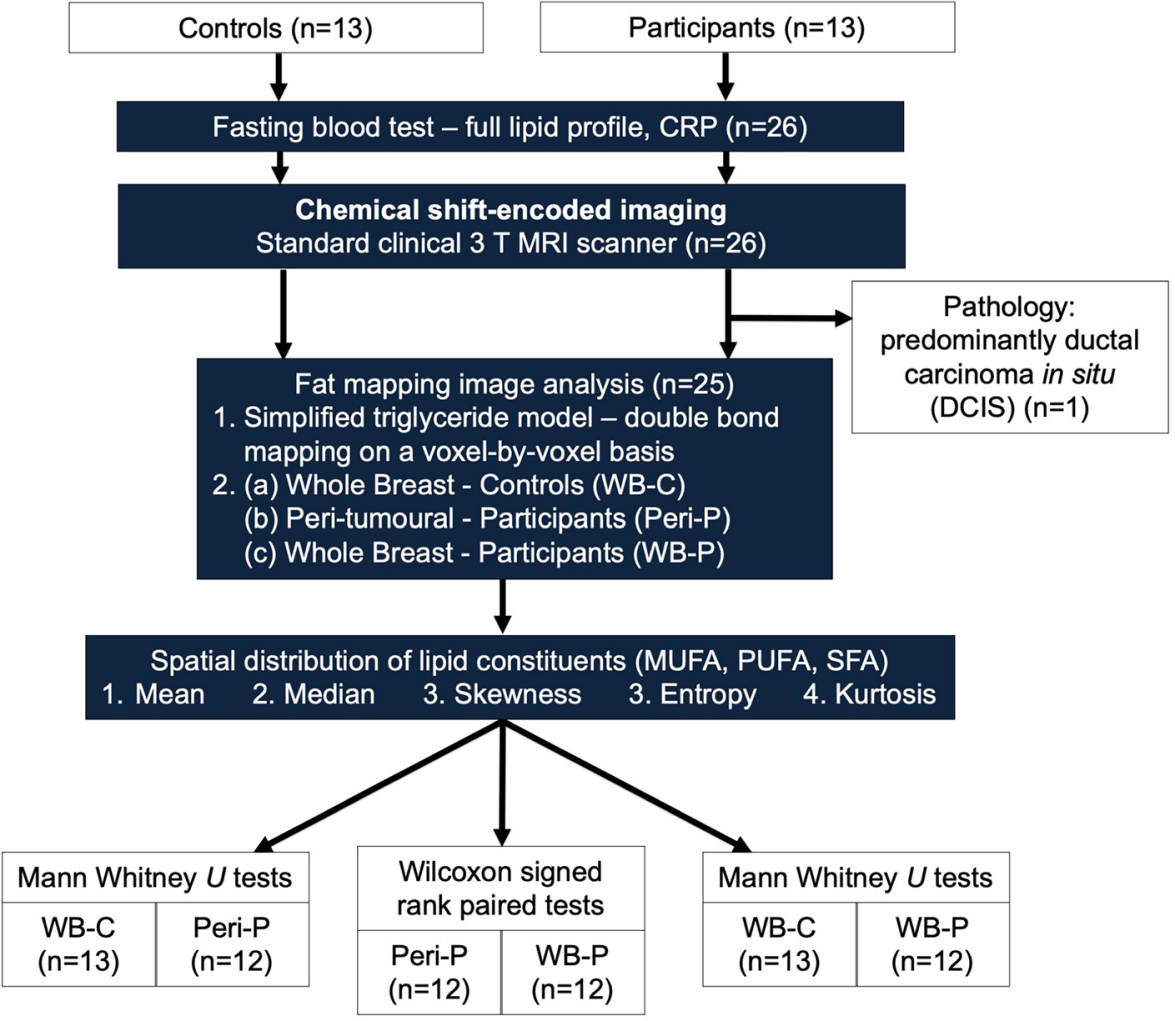
Flowchart of two-group cross sectional research study design. Thirteen postmenopausal patients with oestrogen receptor-positive, invasive ductal carcinoma and 13 age-matched healthy controls were eligible at initial screening and were consented into the study. All participants and controls underwent fasting blood tests on serum full lipid profile (total cholesterol, triglycerides, high density lipoprotein (HDL), low density lipoprotein (LDL) and total cholesterol to HDL ratio) and C-reactive protein (CRP) prior to chemical shift-encoded imaging on a clinical 3T MRI scanner. Fat mapping image analysis was conducted to compute spatial heterogeneity of lipid constituents in the peri-tumoural region (Peri-P) and the whole breast of participants and controls (WB-P, WB-C). Mann Whitney *U* or Wilcoxon signed rank paired statistical tests were subsequently performed to compare values between the locations. *MUFA* monounsaturated fatty acids, *PUFA* polyunsaturated fatty acids, *SFA* saturated fatty acids.

Thirteen postmenopausal participants (mean age, 62 ± [SD] 6 years) with ER + invasive ductal carcinoma and 13 age-matched postmenopausal healthy controls (mean age, 65 ± 5 years) participated in the study. Controls were recruited subsequent to participants with breast cancer and were approximately age-matched with a discrepancy less than 5 years for each participant. Participants undergoing breast conservation surgery, with tumour size larger than 10 mm in diameter on mammography, with no previous malignancies, chemotherapy or radiotherapy prior to surgery were eligible. Participants with diabetes or on statins or cholesterol control drugs were excluded. Controls were not at population risk of breast cancer or at high risk (Fig. 1). The purpose of this study is to understand the role of peri-tumoural lipid composition in postmenopausal participant in preparation for future studies into hormonal therapy, hence breast density was not controlled during recruitment. However, breast density decreases with age^23^ and the current study was conducted in postmenopausal women. Subsequently only one participant and two controls with dense breast were included.

### Clinical procedure

All participants underwent fasting blood tests^24^, and the samples were prepared^25^ and batch-processed^26^ at the Clinical Biochemistry Department of National Health Service Grampian for C-reactive protein (CRP)^27^ and a full lipid profile (total cholesterol, triglycerides, high density lipoprotein (HDL), low density lipoprotein (LDL) and total cholesterol to HDL ratio). Standard clinical histopathological examination was subsequently performed to determine histological tumour size, grade and Nottingham Prognostic Index^28^. Immunostaining was conducted in a single batch for tumour cellular proliferation marker Ki-67^29^ and pro-inflammatory marker CD163 antibody on tumour-associated macrophages^22^ with positive controls in breast tissue, appendix and tonsil and assessed quantitatively by a consultant pathologist (EH) with 20 years of experience in breast pathology. All 13 participants completed MRI scans. The post-surgery pathologic report showed one participant with mostly ductal carcinoma in situ (DCIS); we were unable to define the peri-tumoural region, and this participant was therefore excluded from analyses.

### Lipid composition mapping

All images were acquired on a 3T whole-body clinical MRI scanner (Achieva TX, Philips Healthcare, Best, Netherlands), using a 16-channel breast coil for signal detection and a body coil for uniform transmission. Both T_*1*_- and T_*2*_- weighted anatomical images were acquired from all participants with additional diffusion-weighted images performed in patients to support tumour localisation, however administration of the contrast agent was not included in the study. T_1_-weighted images were acquired using a fast field echo sequence, with echo time of 2.9 ms, repetition time of 5.7 ms, matrix of 192 × 192, pixel size of 1.25 × 1.25 mm^2^, slice thickness of 2 mm. T_2_-weighted images were acquired using a turbo spin echo sequence, with echo time of 60 ms, repetition time of 5000 ms, matrix of 192 × 192, pixel size of 1.25 × 1.25 mm^2^, slice thickness of 2 mm. Diffusion-weighted images were acquired using a pulsed gradient spin echo sequence, with two *b* values of 0 and 800 s/mm^2^, echo time of 60 ms, repetition time of 4000 ms, matrix of 96 × 96, pixel size of 2.5 × 2.5 mm^2^, slice thickness of 4 mm. Lipid composition images were acquired from one breast (diseased breast in patients and left breast in healthy controls) using a two-dimensional CSEI sequence^18,19^ with 174 echoes, an initial echo time of 1.14 ms, echo spacing of 1.14 ms, repetition time of 200 ms, flip angle of 15°, matrix of 64 × 64, pixel size of 3.75 × 3.75 mm^2^, slice thickness of 4 mm and 30 slices. The total acquisition time was 3.5 min.

### Image analysis

**Figure 2:**
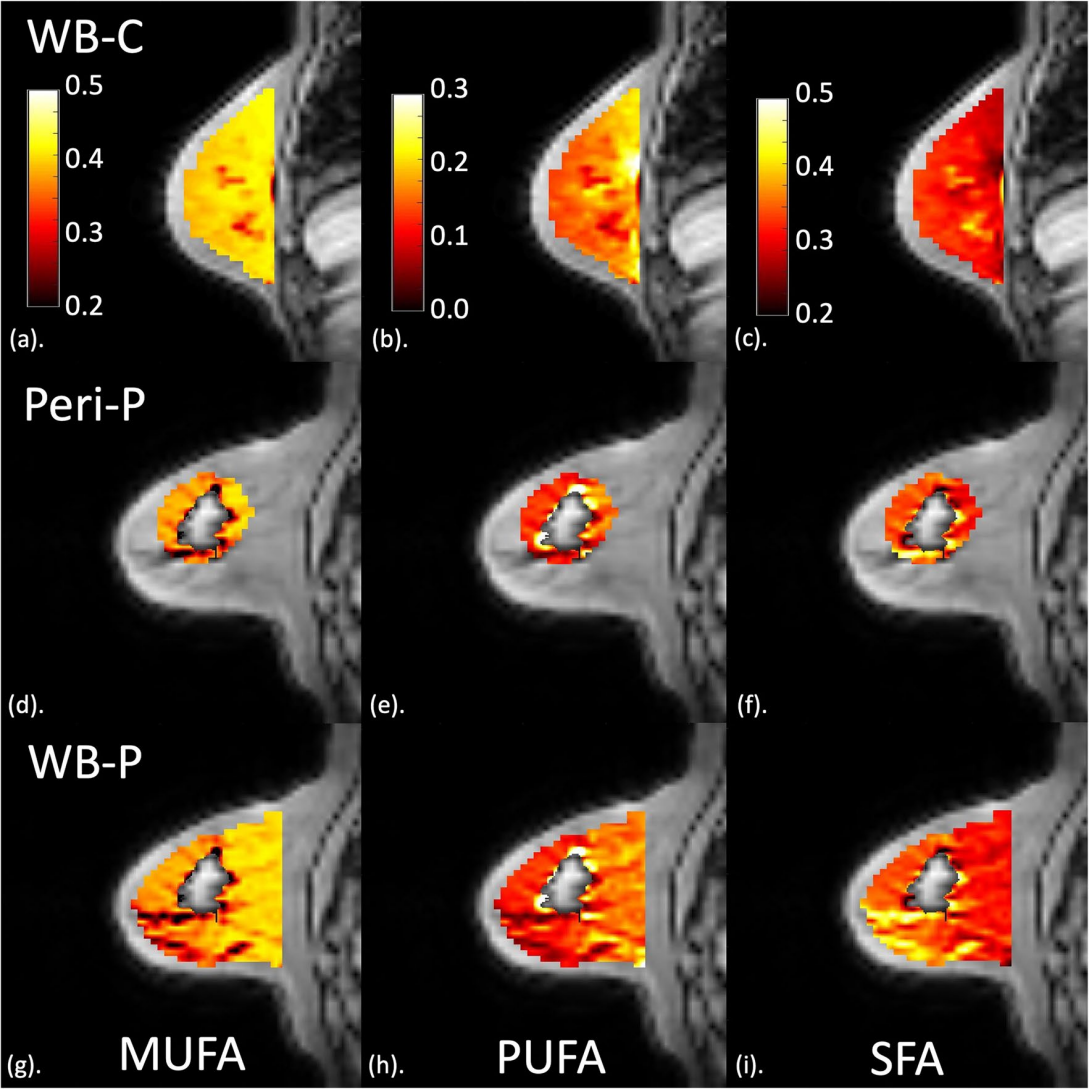
Peri-tumoural and whole breast lipid composition maps, obtained from MRI scans, in a typical participant (65-year-old woman with oestrogen receptor-positive breast cancer) and a control participant (overlaid on anatomical image). (**a**–**c**) MUFA, PUFA and SFA maps in the whole breast of a control participant (WB-C). (**d**–**f**) MUFA, PUFA and SFA maps in the peri-tumoural region of a participant (Peri-P). (**g**–**i**) MUFA, PUFA and SFA maps in the whole breast of a participant (WB-P). For the control participant, the mid-sagittal slice is shown. For the participant, the slice at the greatest dimension of the tumour (central grey area) is shown. *MUFA* monounsaturated fatty acids, *PUFA* polyunsaturated fatty acids, *SFA* saturated fatty acids.

Image analysis was conducted in MATLAB (R2020a, MathWorks Inc., Natick, MA, USA) and ImageJ (v1.52p, National Institute of Health, Bethesda, MD, USA)^30^. The maps showing the number of double bonds in triglycerides were computed from a subset of raw data (first 16 echoes)^18^ prior to the calculation of quantitative maps of MUFA, PUFA and SFA as a percentage of the total amount of lipids^18,19^. Tumour boundary was delineated in all slices of tumour region of interest on the first echo of lipid composition images, with reference to anatomical and diffusion-weighted images, by a consultant radiologist with 15 years of experience in breast MRI examinations. Stringent measures were undertaken during the delineation of tumours and generation of the peri-tumoural regions. The dilation of the tumour region of interest, extraction of the peri-tumoural region and subsequent processing of the lipid composition maps were predefined with automated scripts. Consistent with previous clinical breast imaging studies analysing tumour microenvironment^31,32^, the peri-tumoural region was defined as an outward extension of 15 mm (4 voxels) from the tumour boundary forming a three-dimensional rim around the tumour (Peri-P; see Fig. 2). Our definition of the peri-tumoural region adopted the half way point from published literature, from a thickness of 9–12 mm^32^ to a thickness of 20 mm^31^.

The whole breast was defined as containing only adipose and fibroglandular tissue in controls (WB-C), with further exclusion of the tumour in participants (WB-P). The chest cavity and the subcutaneous fat were removed from images in all participants and controls. Adipose voxels with lipid signals comprising over 60% of the total signal within regions of interest (the peri-tumoural region and the whole breast) were extracted from lipid composition maps for histogram analysis, in accordance with our prior ex vivo study to ensure adequate signal-to-noise ratio for the accuracy of lipid quantification^21^. The spatial heterogeneities (mean, median, skewness, entropy and kurtosis)^11,33^ were subsequently computed based on histogram distribution for each lipid constituent, using the formulae published in our prior study^21^. Skewness indicates the deviation of a distribution from the normal distribution and has both polarity and magnitude. The polarity gives an indication of the relative position of mean and median, while the magnitude estimates the distance between the two. A system with random distribution of a single chemical constituent would assume a Gaussian symmetric bell shape with skewness at zero.

### Sample size and power calculation

The effect size was based on an ex vivo study^21^ considering the values of spatial heterogeneity of lipid composition in peri-tumoural breast adipose tissue. The mean of peri-tumoural MUFA in tumours with low and high grading scores was 0.40 ± 0.01 and 0.38 ± 0.02 respectively. Using standardised mean difference Cohen’s *d*, the estimated effect size was 1.2. The effect size was considered a conservative estimation and anticipated to be higher for comparison between participants and controls. With the average variance at 25% from our previous clinical data, we determined that a sample size of 12 participants per group has 80% power to detect a difference between participants and controls at a two-sided *α* = 0.05 significance level.

### Statistical analysis

All statistical analyses were performed in the *R* software (v3.6.3, The *R* Foundation for Statistical Computing, Vienna, Austria) using ‘stats’ and ‘Hmisc’ packages. Mann Whitney *U* tests were performed to compare the spatial heterogeneity of lipid constituents in the whole breast of participants versus controls (WB-P vs WB-C), and in the peri-tumoural region in participants versus the whole breast in controls (Peri-P vs WB-C). Wilcoxon signed rank paired tests were performed to assess the difference in lipid constituents in the peri-tumoural region compared with the whole breast in participants (Peri-P vs WB-P). The Spearman’s rank correlation coefficients (*r*_*s*_) and 95% confidence intervals (CI) were used to assess correlations between the spatial heterogeneity of each lipid constituent in the peri-tumoural region and tumour size, proliferative activity marker Ki-67 and pro-inflammatory marker CD163 antibody on tumour-associated macrophages. *P* < 0.017 was considered to indicate a statistically significant difference for 3-group comparisons, after Bonferroni correction to avoid Type I errors in the multiplicity of statistical analysis.

### Ethics approval and consent to participate

The study was conducted in accordance with the Declaration of Helsinki and approved by the North of Scotland Research Ethics Service (Identifier: 16/NS/0077), and signed written informed consent was obtained from all participants prior to entry into the study.

## Results

### Participant characteristics

The characteristics of the study participants are shown in Table 1. For the cohort of postmenopausal participants, over 70% (18/25) had scattered fibroglandular tissue, and none had extreme proportion of fibroglandular tissue. There was no evidence of a difference in breast density, body mass index, and serum lipid profile between participants and controls. The histopathological findings in participants are shown in Table 2. The peri-tumoural and the whole breast lipid composition maps from a participant and a typical control are shown in Fig. 2.

**Table 1:** Characteristics of study participants.

Demographic	All (n = 25)	Controls (n = 13)	Participants (n = 12)	*p*-value
Age (years)	63 ± 5	65 ± 5	62 ± 6	.27
Body mass index	27.1 ± 4.2	26.8 ± 4.1	27.3 ± 4.4	.79
Breast density (n)				
Almost entirely fat	4	3	1	.57
Scattered fibroglandular	18	8	10	
Heterogeneous fibroglandular	3	2	1	
Extreme fibroglandular	0	0	0	
Blood serum				
Full lipid profile (mmol/L)				
Total cholesterol	5.6 ± 0.5	5.7 ± 0.4	5.6 ± 0.7	.64
Triglycerides	1.1 ± 0.3	1.2 ± 0.3	1.1 ± 0.3	.64
HDL	1.9 ± 0.4	1.8 ± 0.4	2.0 ± 0.4	.49
LDL	3.2 ± 0.5	3.3 ± 0.3	3.1 ± 0.6	.35
Total cholesterol: HDL	3.1 ± 0.6	3.2 ± 0.6	3.0 ± 0.7	.43
CRP (mg/L)	< 4	< 4	< 4	N/A

Descriptive statistics of controls and participants with breast cancer are shown for each group and the entire cohort. Unless otherwise noted, values are expressed as mean ± SD. Mann Whitney *U* tests and Fisher’s exact test were conducted for group comparisons.

*CRP* C-reactive protein, *HDL* high-density lipoproteins, *LDL* low-density lipoproteins.

**Table 2:** Tumour histology in participants with breast cancer.

Tumour histology	Participants (n = 12)
Tumour Size (cm)	2.4 ± 1.0, (1.2–4.2)
Nottingham prognostic index	4.70 ± 1.05, (3.06–6.66)
Ki-67 (%)	11.1 (8.4), (2.1–40.1)
CD163 (no. of cells/μm^2^)	0.000214 ± 0.000130, (0.000018–0.000370)
Histology grade	
II	4 (33.3%)
III	8 (66.7%)
Lymphovascular invasion	
Negative	4 (33.3%)
Positive	8 (66.7%)
Lymph node involvement	
Negative	6 (50.0%)
Positive	6 (50.0%)
Molecular subtypes	
Luminal A	5 (41.7%)
Luminal B-HER2 negative	4 (33.3%)
Luminal B-HER2 positive	3 (25.0%)
Triple Negative	0 (0.0%)

Histopathological findings for participants with breast cancer are shown, with quantitative data expressed as mean ± SD or median (interquartile range) and qualitative data expressed as number of positive observations. Range and group percentage are also shown respectively. Nottingham Prognostic Index is calculated using the formula: Grade (1– 3) + Node (1–3; 1: negative nodes, 2: 1–3 positive nodes, 3: > 3 positive nodes) + Size of tumour (in centimetre × 0.2).

*HER2* human epidermal growth factor receptor 2.

### Peri-tumoural region versus whole breast in participants

**Figure 3:**
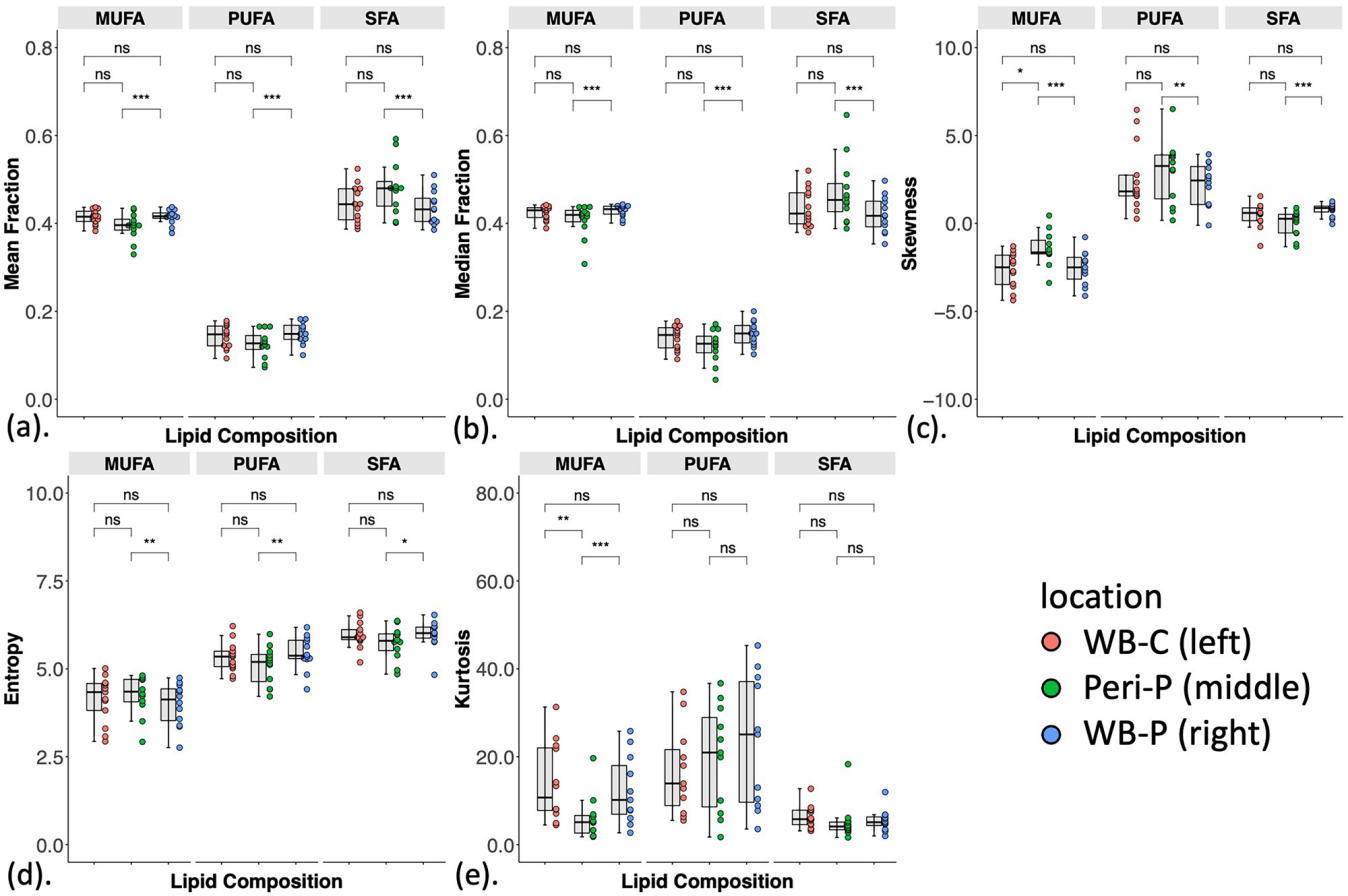
The group difference in (**a**) mean, (**b**) median, (**c**) skewness, (**d**) entropy and (**e**) kurtosis of MUFA, PUFA, SFA in the whole breast of control participants (WB-C) (n = 13), the peri-tumoural region (Peri-P) (n = 12) and the whole breast of participants (WB-P) (n = 12). Each dot represents a peri-tumoural or whole breast mean fraction or spatial heterogeneity, and the dots are organised in three columns corresponding to locations. All spatial heterogeneities are shown in boxplots to indicate the minimum, 25th percentile, median, 75th percentile and maximum. Mann Whitney *U* (participants versus control participants) and Wilcoxon signed rank paired (within participants) tests were performed between the groups. Statistical significant *p* values are marked by *(< .017 after Bonferroni correction for multiple comparisons), **(< .01), ***(< .001). ‘ns’: not significant. *MUFA* monounsaturated fatty acids, *PUFA* polyunsaturated fatty acids, *SFA* saturated fatty acids.

There were significant differences in mean, median, skewness, and entropy of all lipid constituents and kurtosis of MUFA (Fig. 3, Table 3) between the peri-tumoural region and whole breast in participants. For MUFA, there was a lower mean (*p* < .001), lower median (*p* < .001), a negative skewness with lower magnitude (*p* = .001), higher entropy (*p* = .007), and lower kurtosis (*p* = .001) in the peri-tumoural region (Fig. 3, Table 3). For PUFA, there was a lower mean (*p* < .001), lower median (*p* < .001), a positive skewness with higher magnitude (*p* = .005), and lower entropy (*p* = .002) in the peri-tumoural region, but no evidence of a difference in kurtosis (Fig. 3, Table 3). For SFA, there was a higher mean (*p* < .001), higher median (*p* < .001), a positive skewness with lower magnitude (*p* < .001), and lower entropy (*p* = .012) in the peri-tumoural region, but no evidence of a difference in kurtosis (Fig. 3, Table 3).

**Table 3:** Peri-tumoural and whole breast monounsaturated, polyunsaturated and saturated fatty acids (MUFA, PUFA, SFA).

Lipid	Spatial heterogeneity measures	Controls (n = 13)	Participants (n = 12)		Peri-P versus WB-P	Peri-P versus WB-C	WB-P versus WB-C
		Whole breast (WB-C)	Peri-tumoural (Peri-P)	Whole breast (WB-P)	*p*	*p*	*p*
		median (IQR)	Median (IQR)	median (IQR)			
MUFA	Mean	0.42 (0.02)	0.40 (0.02)	0.42 (0.01)	< .001*	.06	.94
	Median	0.43 (0.02)	0.42 (0.02)	0.43 (0.02)	< .001*	.23	.47
	Skewness	− 2.50 (1.67)	− 1.65 (0.77)	− 2.50 (1.24)	.001*	.01*	.98
	Entropy	4.34 (0.77)	4.35 (0.64)	4.13 (0.90)	.007*	.73	.61
	Kurtosis	10.73 (14.24)	5.13 (3.99)	10.18 (11.08)	.001*	.009*	.65
PUFA	Mean	0.14 (0.04)	0.13 (0.03)	0.15 (0.03)	< .001*	.23	.50
	Median	0.15 (0.05)	0.13 (0.02)	0.15 (0.04)	< .001*	.29	.47
	Skewness	1.82 (1.19)	3.27 (2.49)	2.44 (2.17)	.005*	.54	.94
	Entropy	5.35 (0.44)	5.20 (0.77)	5.37 (0.52)	.002*	.41	.47
	Kurtosis	13.92 (12.75)	20.95 (20.34)	25.08 (27.46)	.24	.52	.33
SFA	Mean	0.44 (0.07)	0.48 (0.06)	0.43 (0.05)	< .001*	.11	.69
	Median	0.42 (0.07)	0.45 (0.06)	0.42 (0.05)	< .001*	.29	.47
	Skewness	0.59 (0.73)	0.27 (1.06)	0.88 (0.34)	< .001*	.08	.23
	Entropy	5.90 (0.29)	5.80 (0.48)	6.02 (0.31)	.012*	.25	.54
	Kurtosis	5.80 (3.27)	4.14 (1.77)	5.12 (1.96)	.20	.08	.44

The mean, median, skewness, entropy and kurtosis of lipid constituents were compared between participants and healthy controls. Mann Whitney *U* tests were conducted for comparisons ‘Peri-P versus WB-C’ and ‘WB-P versus WB-C’. Wilcoxon signed rank paired tests were conducted for comparison ‘Peri-P versus WB-P’.

*Denotes a statistically significant difference after Bonferroni correction for multiple comparisons (*p* < .017).

### Peri-tumoural region in participants versus whole breast in controls

For MUFA, there was a negative skewness with lower magnitude (*p* = .01) and lower kurtosis (*p* = .009) in the peri-tumoural region compared with the whole breast in controls (Fig. 3, Table 3). There was no evidence of differences in other spatial heterogeneities of MUFA, nor in any spatial heterogeneities of PUFA or SFA.

### Whole breast in participants versus whole breast in controls

When comparing the whole breast of participants with that of controls, we found there was no evidence of differences in mean, median, skewness, entropy and kurtosis of any lipid constituents (Fig. 3, Table 3).

### Correlation between peri-tumoural region and Ki-67 and CD163

**Figure 4:**
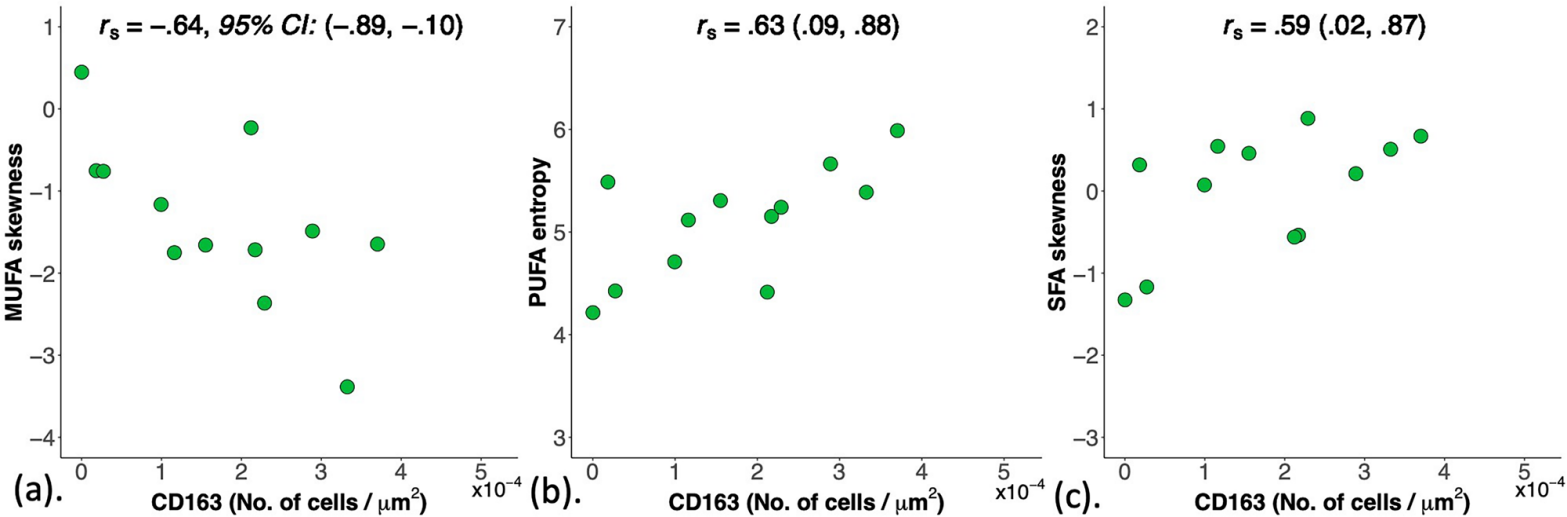
Scatter plots showing correlations of (**a**) MUFA skewness (n = 12), (**b**) PUFA entropy (n = 12) and (**c**) SFA skewness (n = 12) with CD163 antibody on tumour-associated macrophages. The Spearman’s rank correlation coefficients (*r*_*s*_) and 95% confidence intervals (CI) are shown for each plot. *MUFA* monounsaturated fatty acids, *PUFA* polyunsaturated fatty acids, *SFA* saturated fatty acids.

MUFA skewness and kurtosis were not correlated with Ki-67 (Table 4). MUFA skewness had a moderate negative correlation (*p* = .03, Fig. 4a, Table 4) with CD163. PUFA entropy had a moderate positive correlation (*p* = .03, Fig. 4b, Table 4) with CD163. SFA skewness had a moderate positive correlation (*p* = .04, Fig. 4c, Table 4) with CD163.

**Table 4:** Correlations between peri-tumoural monounsaturated, polyunsaturated and saturated fatty acids (MUFA, PUFA, SFA) in participants and tumour size and histopathological markers.

Lipid	Spatial heterogeneity measures	Participants (n = 12)	Tumour size		Ki-67		CD-163	
		Peri-tumoural (Peri-P) Median (IQR)	*r*_*s*_	95% CI	*r*_*s*_	95% CI	*r*_*s*_	95% CI
MUFA	Mean	0.40 (0.02)	− .34	(− .77, .29)	.25	(− .27, .78)	.52	(− .08, .84)
	Median	0.42 (0.02)	− .27	(− .73, .36)	.35	(− .34, .74)	.45	(− .17, .81)
	Skewness	− 1.65 (0.77)	.10	(− .50, .64)	− .26	(− .75, .33)	− .64*	(− .89, − .10)
	Entropy	4.35 (0.64)	.39	(− .24, .79)	.06	(− .54, .61)	.04	(− .55, .60)
	Kurtosis	5.13 (3.99)	− .09	(− .63, .51)	.31	(− .32, .75)	.55	(− .04, .85)
PUFA	Mean	0.13 (0.03)	− .34	(− .77, .29)	.40	(− .23, .79)	.48	(− .14, .82)
	Median	0.13 (0.02)	− .24	(− .72, .39)	.30	(− .34, .74)	.45	(− .17, .81)
	Skewness	3.27 (2.49)	.03	(− .55, .59)	− .29	(− .74, .34)	− .45	(− .81, .17)
	Entropy	5.20 (0.77)	.15	(− .46, .67)	.47	(− .14, .82)	.63*	(.09, .88)
	Kurtosis	20.95 (20.34)	.11	(− .50, .64)	− .20	(− .69, .42)	− .36	(− .78, .27)
SFA	Mean	0.48 (0.06)	.34	(− .29, .77)	− .36	(− .78, .27)	− .52	(− .84, .07)
	Median	0.45 (0.06)	.32	(− .31, .76)	− .30	(− .74, .34)	− .45	(− .81, .17)
	Skewness	0.27 (1.06)	− .15	(− .66, .47)	.37	(− .26 .78)	.59*	(.02, .87)
	Entropy	5.80 (0.48)	.26	(− .37, .73)	.38	(− .25, .78)	.55	(− .03, .86)
	Kurtosis	4.14 (1.77)	− .27	(− .73, .36)	− .01	(− .58, .57)	− .04	(− .60, .55)

The Spearman’s rank correlation coefficients (*r*_*s*_) and 95% confidence intervals (CI) for peri-tumoural lipid constituents with tumour size, proliferative activity marker Ki-67 and pro-inflammatory marker CD163 antibody on tumour-associated macrophages are shown.

*Denotes a statistically significant correlation since the confidence interval does not contain the null hypothesis value (0).

### Correlation between peri-tumoural region and tumour size

The correlation between peri-tumoural lipid composition and tumour size is shown in Table 4; all correlation was nonsignificant.

## Discussion

In this in vivo investigation of peri-tumoural spatial heterogeneity of lipid composition in women with breast cancer, lipid composition maps acquired from MRI scans showed significant differences in mean, median, skewness and entropy of all lipid constituents between the peri-tumoural region and the whole breast in participants. The results demonstrated that the quantity and spatial distribution of peri-tumoural lipid composition were altered in postmenopausal women with ER + breast cancer, providing potential clinical markers to further the evidence from cellular and ex vivo studies. The difference in peri-tumoural MUFA in comparison to the whole breast in controls indicated the potential critical role of MUFA during tumour initiation. The degree of lipid deregulation was associated with CD163 antibody on tumour-associated macrophages, indicating the deployment of lipid in the peri-tumoural region as a dynamic process of inflammation during tumour progression.

Since MUFA showed significant differences in all the measures of peri-tumoural spatial heterogeneity compared with the whole breast in participants, MUFA might be the primary end product in lipid deregulation in the tumour microenvironment. Specifically, the lower mean and median of peri-tumoural MUFA compared with the whole breast suggests a tumour induced local reduction in MUFA. Both the polarity and magnitude of skewness have respective physical meaning in the context of this work. In the scenario of two pools (healthy and diseased), the polarity would indicate the dominant pool, while the magnitude indicating the balance between the two. We hence point out the polarity and magnitude in the results to allow a more direct conceptualisation of the underlying biological processes. The negative skewness for MUFA in both participants and controls indicates the distribution peaking at a higher value and extending further into lower values, while the lower magnitude of skewness in the peri-tumoural region indicates a more balanced distribution centred at a lower mean than the whole breast. Therefore, the tumour may induce regional reduction of MUFA in the breast within the adipose tissue adjacent to the tumour. The lower kurtosis and higher entropy of MUFA in the peri-tumoural region compared with the whole breast in participants indicate a more highly concentrated non-random distribution with less outliers and a more heterogeneous distribution, respectively^11,33^. It has been shown that there was a greater activity of the stearoyl-coenzyme-A desaturase-1 in ER + breast cancer cells than in ER- cells to accelerate MUFA production in breast cancer adipose tissues and generate phosphatidylcholine for membrane formation^34^. Thus, both the incorporation of MUFA into the tumour core to support membrane synthesis^35^ and infiltration of breast carcinoma cells into peri-tumoural adipocytes^36^ may lead to widespread MUFA reduction and heterogeneous spread^37^. The negative association between MUFA skewness in the peri-tumoural region and the CD163 antibody on tumour-associated macrophages suggests that spatial distribution of MUFA might impact pro-inflammatory activities.

There were significant differences in all spatial heterogeneity measures of PUFA, except for kurtosis; therefore, PUFA might be the secondary end product in lipid deregulation in the peri-tumoural region. The lower mean and median of PUFA in the peri-tumoural region compared with the whole breast in participants suggests increased uptake of exogenous PUFA to sustain the higher demand from tumour proliferation^38^ and pro-inflammatory eicosanoid synthesis^36^. The positive skewness across groups indicates the distribution peaking at a lower value and extending into higher values, while the higher magnitude of skewness in the peri-tumoural region indicates more unbalanced distribution with a lower mean than the whole breast. The lower entropy of PUFA in the peri-tumoural region compared with the whole breast in participants indicates a more homogeneous distribution. PUFA remodels lipid droplets adjacent to the endoplasmic reticulum nuclear membrane to perturb the extracellular matrix architecture in MCF-7 (Luminal A, ER +) breast cancer cell lines with comparable impact on MDA-MB-231 (TNBC, ER-) cells^39^. The association between PUFA entropy in the peri-tumoural region with the CD163 antibody on tumour-associated macrophages suggests that PUFA may be involved in pro-inflammatory activities^36^, and may therefore be a critical surrogate for inflammation.

The elevated mean and median of SFA in the peri-tumoural region compared with the whole breast in participants suggests an extrusion of SFA from the tumour, with subsequent reduction in lipotoxicity and increased membrane rigidity for enhanced survival advantage^40^, as well as resistance to cancer therapies^41^. The positive skewness for SFA across groups indicates the distribution peaking at a lower value and extending into higher values, while the lower magnitude of skewness in the peri-tumoural region indicates a more balanced distribution centred at a higher mean than the whole breast. The lower entropy of SFA in the peri-tumoural region compared with the whole breast in participants indicates a more homogeneous distribution, potentially due to the uniform export of SFA across the tumour boundary^21,41^. An increased release of SFA in the tumour microenvironment stimulates macrophages to manufacture pro-inflammatory mediators, including cyclooxygenase-2 and tumour necrosis factor alpha^42^, leading to enhanced aromatase expression in adipocytes and sustained oestrogen biosynthesis for tumour progression^43,44^. However, MCF-7 cells are less sensitive to SFA modulation during nuclear membrane remodeling compared with MDA-MB-231 cells^39^. The association between SFA skewness in the peri-tumoural region and the CD163 antibody on tumour-associated macrophages suggests that SFA export might not only alleviate cell apoptosis but further support pro-inflammatory activities^41^. Therefore, the spatial heterogeneity of peri-tumoural SFA may reflect underlying inflammation, with the potential to become biomarkers for early diagnosis and response to therapy since an elevated level of macrophages is associated with higher recurrence and poorer prognosis^45^.

This prospective study adopted strict inclusion criteria to minimise the impact of confounding factors and answer a focused research question. All participants had invasive ductal carcinoma, with Luminal A or Luminal B ER + breast cancer, stage 2A or 2B. Given that less than 3 groups were analysed, correlation analyses were not conducted on peri-tumoural lipid composition within tumour type or stage categories. Lipid composition maps were acquired from a single breast to ensure optimal shimming and high image quality, and the classification of the peri-tumoural region in participants was well-defined, yielding consistency in comparisons. A comparison between the peri-tumoural region and the whole breast in controls answered a secondary research question on the potential of aberration in lipid composition as a clinical research tool for early diagnosis, with the ultimate aim to detect anomaly in the whole breast of women with a high risk of breast cancer.

Our study had limitations. First, this was a prospective study with a small cohort, and future large cohort studies are required to demonstrate the effectiveness of lipid composition as biomarkers for early diagnosis. Second, the investigation was a prospective study with recruitment of consecutive participants, with one participant and two controls with dense breast. Future cohort to focus on women with high density of the breast will be valuable. Third, this was not an interventional study, and the contribution of small but significant changes in heterogeneity of lipid composition will require future interventional studies to ascertain the clinical value of the imaging markers for determining the efficacy of hormonal therapy in postmenopausal patients with ER + breast cancer^46^. Fourth, a comparison between the peri-tumoural region and the whole breast in controls might be impacted by extreme values, although represented a tangible step forward in comparison to loosely defined regions of interest^6,7^. A refined reference to account for specific breast tissue and anatomy other than the whole breast may be devised in a future study to enhance our understanding of the role of lipids in breast cancer.

## Conclusion

There was a lower MUFA and PUFA while a higher SFA, and a higher heterogeneity of MUFA while a lower heterogeneity of PUFA and SFA, in the peri-tumoural region in comparison with the whole breast tissue. The degree of lipid deregulation was associated with inflammation as indicated by CD163 antibody on macrophages, serving as potential marker for early diagnosis and response to therapy.

## Data Availability

The datasets used and analysed during the current study are available from the corresponding author on reasonable request.

## Abbreviations

CSEI: Chemical shift-encoded imaging
ER +: Oestrogen receptor-positive
MUFA: Monounsaturated fatty acids
PUFA: Polyunsaturated fatty acids
SFA: Saturated fatty acids
