# Author Correction: Spatial heterogeneity of peri-tumoural lipid composition in postmenopausal patients with oestrogen receptor positive breast cancer

**DOI:** 10.1038/s41598-024-60477-w

**Published:** 2024-05-06

**Authors:** Sai Man Cheung, Kwok-Shing Chan, Wenshu Zhou, Ehab Husain, Tanja Gagliardi, Yazan Masannat, Jiabao He

**Affiliations:** 1 School of Medicine, Medical Sciences and Nutrition, University of Aberdeen, Aberdeen, UK; 2 Athinoula A. Martinos Center for Biomedical Imaging, Charlestown, MA USA; 3 Department of Radiology, Harvard Medical School, Boston, MA USA; 4 Department of Pathology, Aberdeen Royal Infirmary, Aberdeen, UK; 5 Department of Radiology, Royal Marsden Hospital, London, UK; 6 Broomfield Breast Unit, Broomfield Hospital, Mid and South Essex NHS Trust, Chelmsford, UK; 7 London Breast Institute, Princess Grace Hospital, London, UK; 8 Faculty of Medical Sciences, Newcastle Magnetic Resonance Centre, Translational and Clinical Research Institute, Newcastle University, Newcastle upon Tyne, UK

Correction to: *Scientific Reports* 10.1038/s41598-024-55458-y, published online 26 February 2024

In the original version of this Article, Jiabao He was omitted as a corresponding author. Correspondence and requests for materials should also be addressed to jiabao.he@newcastle.ac.uk.

The original Article has been corrected.

